# An application of multi-criteria decision-making approach to sustainable drug shortages management: evidence from a developing country

**DOI:** 10.1186/s40780-021-00200-3

**Published:** 2021-04-02

**Authors:** Asiye Moosivand, Maryam Rangchian, Leila Zarei, Farzad Peiravian, Gholamhossein Mehralian, Hesameddin Sharifnia

**Affiliations:** 1 Department of Pharmacoeconomics and Pharma Management, Shahid Beheshti University of Medical Sciences, Tehran, Iran; 2 School of Pharmacy, Hamadan University of Medical Sciences, Hamadan, Iran; 3 Health Policy Research Center, Institute of Health, Shiraz University of Medical Sciences, Shiraz, Fars Iran; 4 Department of Clinical Epidemiology, Faculty of Medicine, AJA University of Medical Sciences, Tehran, Iran

**Keywords:** Drug shortage, Multi-criteria decision-making, Pharmaceutical policy, Supply chain management, Iran

## Abstract

**Background:**

Drug shortage is a significant public health problem, especially for drugs related to life threatening conditions. Almost all countries affected by variety of supply problems and spent a considerable amount of time and resources responding to shortage. The aim of present study is to determine and prioritize strategies to achieve best solutions for these considerable healthcare system challenges and to evaluate this strategies base on practical criteria.

**Methods:**

To achieve the study objectives, the research was conducted in two phases. Determining of the strategies to control drug shortage, and comprehensive assessments of priority of possible strategies. For each phase, a self-design questionnaire was developed. The five main managerial strategies dimensions including: regulatory, financial, supply chain, information system and policy-making were set out. Forty-five alternatives were elicited from literature, and were evaluated and trimmed to 37 strategies based on experts’ opinion. The Multiple criteria decision-making (MCDM) methods were applied in second phase. Five important criteria including cost, time, labor, compliance with law and culture were weighed by Analytic Hierarchy Process (AHP) technique. Then, 37 alternatives have been rated base on the five criteria on the Technique for Order of Preference by Similarity to Ideal Solution (TOPSIS) technique.

**Results:**

“Creating integrated Supply chain information system to manage medicines inventory in the country”, “Creating and using the databases to predict the shortage of medicines”, “Using track and trace system” are alternatives 20th, 24th and 25th, which related to supply chain (SC) and information system (IS) dimensions have higher priority in the experts’ point of view. The results show IS dimension has 100 percentage of priority; following that policy and supply chain have higher priority, respectively.

**Conclusion:**

Health systems rely on consistent supplying of pharmaceuticals to support patient care. The results show that information system, policy-making and supply chain are in the top-ranking priorities. Warning system needs to be improved to the advance system via better collaboration with stakeholders, publish precise and explicit national guidelines for drug shortage management, enforce the guidelines, and improve Iran FDA’s pharmaceutical market control capability.

## Introduction

Drug shortages are a well-established, rapidly growing hallmark of current economic conditions [1]. Such scarcity, especially the shortage in drugs that are used to treat life-threatening conditions, is a significant public health problem that deserves concerted attention from governments and pharmaceutical industries [2, 3]; the substitution of safe and effective medications with alternative drugs or dosage forms compromise or delay medical procedures or cause medication errors [4, 5]. In these situations, irreparable damage and premature death have sometimes occurred [6].

Several studies have identified drug shortages as a complex global challenge [7, 8]. All healthcare systems have established plans for practice, which are refined in preparation for an unexpected crisis, such as a drug shortage [7, 9]. Nevertheless, shortfalls in medicine supply continue to be an enormous concern in many countries, such as the United States [3, 10–12], Europe [13–15], Australia [16], Germany [17], and Canada [18]. Given that adopting the most effective strategies plays a pivotal role in controlling drug shortages, this study was conducted to determine and prioritize strategies and procedures for achieving the best solutions to this considerable healthcare system challenge and to evaluate the strategies on the basis of practical criteria.

## Research background

### Drug shortage

The definition of what a drug shortage is can vary depending on national drug policies, intended time period, and other effective factors. According to the qualitative study conducted by Weerdt [15], the most common definitions of drug shortage in scientific documents are those proposed by the American Society of Hospital Pharmacies (ASHP) and the Food and Drug Administration (FDA). The ASHP defined a drug shortage as “a supply issue that affects how the pharmacy prepares or dispenses a drug product or influences patient care when prescribers must use an alternative agent” [19]. The FDA defined it as “a situation in which the total supply of all clinically interchangeable versions of a drug is inadequate to meet current or projected demand at the user level” [3].

Drug shortages have been the topic of interest in a vast number of studies in the West, particularly the US [12, 20–23]. US-based studies investigated the lack of uniformity in the manner by which drug shortages are addressed as well as the scope, causes, and effects of drug shortages. In recent years, an increased volume of research concerning drug shortages in Europe has been published, with researchers attempting to gauge the scope and causes of the problem [13–15, 24, 25]. Many studies focused on the shortage of medically necessary products that exert a significant effect on public health or the scarcity of more common drugs [20, 26–29], and others delved into evidence-based practice in times of drug shortage [19, 30].

A review of published and unpublished information on drug shortages showed that drug shortages are rooted in economic, legal, regulatory, policy, and clinical decisions that are deeply interconnected [8, 23, 31–33].. Changes in drug supply can alter the manner by which medications are prepared in pharmacies and administered to patients [23]. Although drug shortages have been steadily rising every year since 2006, it has only recently received media attention [34].

Escalating drug shortages are caused by various factors, including difficulties in acquiring raw materials, manufacturing problems, regulatory issues, business decisions, and many other disturbances within the supply chain [16, 23, 35]. Heretofore, numerous solutions had been proposed to manage drug shortages [10, 36, 37], but no single or simple solution that can resolve this social problem has been identified [12, 31]. Efforts to address the problem will need to be multifaceted, sustained over the long term, and grounded in the engagement of different affected stakeholders [8].

### Strategies for managing Drug shortages

A number of strategies have been formulated to enable healthcare systems to prevent, mitigate, and respond to drug shortages. Although predicting or preparing for every drug shortage is impossible, the problem can be minimized through data collection via a routine market-monitoring program. A lack of advance warning of impending shortage hinders the allocation of adequate time to systematic and appropriate communication among parties responsible for addressing shortages [6]. Some studies recommended contacting regional and local drug information centers for assistance in assembling a list of alternatives and supporting literature [12]. Establishing clear procedures and guidelines for managing drug shortages is also essential [10, 23]. To sum up, appropriation information collection, extensive collaboration, timely communication, and clear guidelines are the critical elements of an effective drug shortage management plan [12, 23]. In terms of responding to drug shortages, finding the cause of the shortage can often provide clues about its duration; these steps must be determined early in the management process [22, 38]. A comprehensive evaluation of how the drug shortage will affect patient care should then be conducted [12, 19, 39]. For example, a threat analysis based on a shortage’s expected duration and an assessment of current inventory and usage patterns can be carried out to determine the potential consequences of the shortage [19].

Effective management necessitates the sequestration of internal medication supplies, which means locating all medications that are available within a healthcare system [39]. All options should be evaluated, including alternative therapies, contract compounding, priority-driven dispensing, and rationing [1]. The strategies should be re-evaluated regularly because circumstances will likely change during a shortage [3].

During a severe shortage, prioritizing patients is sometimes necessary and is one of the most challenging components of shortage management. In addition, a cooperative spirit between regulatory body and drug manufacturers must be developed, with constant sharing of regulatory standards, to enable manufacturing to be incrementally upgraded and prevent disruptions to drug production [40].

### Drug shortage in Iran

In Iran, a severe drug shortage occurred from 2010 to 2012 [41]. Similar to other regulatory agencies around the world, the Iranian healthcare system collaborated with manufacturers and distributors to prevent and mitigate these shortages, yet about 370 items remained in the drug shortage list in 2012. This was an alarming situation given that drug shortage lists typically contain only about 30 to 40 items [42].

The IFDA’s definition of shortage is similar to that the FDA has defined. Therefore, based on IFDA policies, the shortage in specific brands with available generic alternatives are not considered as “shortage”. The IFDA conducts a formal and periodic market monitoring program to improve the ability of Iran’s healthcare system to predict drug shortages [43]. The organization also implements intensive inventory control by holding quarterly formal meetings among the local producers, importers, and distributers/wholesalers primarily involved in the medicine supply chain [44]. The participation of all stakeholders in these meetings helps them keep inventory costs at a minimum while ensuring the availability of medicines. Some impending shortages in the country were recognized through the consideration of the results of the formal meetings, future demand with reference to historical consumption, and some other previously forecast lack of supply. Unfortunately, some suppliers do not abide by the IFDA’s regulations and, at certain times, the intensive inventory control meetings are insufficiently effective. As a consequence of failure to follow predetermined supply programs, drug shortages occur [42]. Another measure taken by the IFDA is the proactive inventory program, which functions as an early warning system and as an avenue for the collection of shortage data through the IFDA’s Information Center of Medicine, medical science universities, and some referral pharmacies all over Iran. As part of the program, a list of drugs that are in short supply is provided regularly to all pharmaceutical companies. The list is prepared on the basis of the International Nonproprietary Names (INN) of pharmaceutical products. Substantial data on branded medicines are available from the Information Center of Medicine, but a lack of supply of brands with available generic alternatives is not considered “shortage.” Registered emergency pharmaceutical companies are responsible for supplying medicines in shortage from foreign wholesalers registered in the IFDA [45]. Finally, during a shortage, the IFDA implements a distribution policy, which stipulates that scarce drugs should be distributed through special pharmacies such as universities’ pharmacies for the effective monitoring of medications and prioritization of patients in some cases [42].

Despite the headway achieved by the IFDA, some weaknesses in the risk assessment and management approaches of Iran impede the enactment of pharmaceutical policies and strategies. For instance, the management of future drug shortages is confronted with difficulties and stakeholder collaborations are weakened because of a lack of shortage experience documentation that identifies the causes and patterns of scarcity. Weaknesses in the strategic supply chain management (SCM) approaches such as supplier monopoly or dependence on imported raw or packaging materials can present challenges to local pharmaceutical companies that manufacture finished products. Two other signs of an inefficient SCM approach by the IFDA include: 1) lack of planning and strategic drug stocking, especially for essential medicines that can facilitate drug shortage management, and 2) national centers in charge of monitoring physicians’ prescription behaviors and providing scientific medicinal information to the community, have not been formally assigned a sufficient role in the drug shortage management scheme of Iran.

## Research methodology

### Instruments and measures

Given the special features of each healthcare system, policy makers should endeavor to understand which actions should be considered and which should be regarded high priority. Illuminating this issue necessitates the use of a previously published holistic questionnaire that enables experts to identify and prioritize strategies on the basis of core measures.

To achieve the research objectives, this study was conducted in two phases. The first involved determining strategies for controlling drug shortages, and the second entailed a comprehensive assessment of priority alternative strategies, which were ascertained in the previous phase. For the first phase, a self-designed questionnaire was developed on the basis of an extensive literature review. At first, 45 alternatives were elicited from literature. Then they were evaluated and trimmed based on 12 experts’ opinion. Finally, 37 alternatives and five dimensions of principal managerial strategies, namely, the regulatory, financial, supply chain, information system, and policy making dimensions, were identified. A five-point Likert scale ranging from 1 (“strongly disagree”) to 5 (“strongly agree”) was used to score the items. The dimensions and their related questions determined in phase one are shown in Table 1.

**Table 1:** Dimensions and related questions

Dimensions	Questions (Alternatives)
**Regulatory**	A1: Preparation and periodic review of nation drug list. A2: Forming the formulary committee to modify unnecessary drugs from list. A3: Improving drug shortage monitoring department in IFDA. A4: Formulating drug and raw material registration processes based on health system priorities. A5: Developing the protocols for each part of SC. A6: Legislation for information sharing in SC. A7: Prosecution of the companies causing drug shortage due to not notifying on time. A8: Monitoring the punishments for illegal drug import and export. A9: Clarifying and stabilizing rules and procedures. A10: Technical and qualitative monitoring of production lines to prevent a sudden stop in production.
**Financial**	A11: Prioritize supporting the entry of raw materials and essential drugs, according to the list of essential drugs. A12: Using different competitive pricing for imported drugs. A13: Reinforcement of cooperation and coordination with other relevant organizations.
**Supply chain**	A14: Planning for producing the raw material and finished products imported based on SC information. A15: Pharmaceutical companies’ timely notification to the IFDA about the recall or problems in production and import of products. A16: Creating a competitive environment for imports. A17: Restructuring emergency pharmaceutical companies to meet the real needs of pharmaceutical market. A18: Establishment of an integrated and capillary distribution system based on supply chain information. A19: Improving the delivery system to the remote areas. A20: Using track and trace system. A21: Encouraging patients to use alternative drugs available in the drug shortage by physicians. A22: Commitment of physicians to prescribe drugs based on national formulary. A23: Physicians should be up to date and alert about the availability of the drugs in market and the alternatives of medicines in shortage.
**IS**	A24: Creating an integrated supply chain information system to manage medicines inventory. A25: Creating and using the databases to predict the shortage of medicines.
**Policy-making**	A26: Taking out the OTC drugs from insurance coverage and allocate their budget to essential drugs. A27: Preparing alternative medicine list by the expert committee for crisis condition. A28: Reducing medicine waste by promoting rational use. A29: Saving the strategic supply of essential medicines and keeping them up to date in macro level. A30: Managing the allocation of drugs based on diseases priority in times of crisis. A31: Adopting policies to build partnerships between all components of the supply chain and health services for the establishment of an integrated management. A32: Recording strategies taken for drug shortage management and the reasons for their success or failure for use in future events. A33: Adopting policies to increase the competitiveness of domestic pharmaceutical industry. A34: Adopting policies to prevent the black market and drugs smuggling. A35: Creating pharmaceutical industry think-tank to predict the effects of various economic and political factors on the pharmaceutical market. A36: Actual value-based pricing and improving insurance coverage. A37: Decreasing the effect of governmental authority in the field of production and import of medicine

Multiple-criteria decision-making (MCDM) methods were applied in the second phase because they are very powerful tools that are widely used to address unstructured problems whose resolution is underlain by multiple and potentially conflicting objectives [46].

First, the importance and weight of decision attributes should be determined. Thereby, analytic hierarchy process (AHP) was applied in this part.

The AHP method dates back to the early 1970s, during which it was used for resource allocation and planning in the military; it is designed to reveal the manner by which people actually think (Saaty, 1994). In using the AHP to model a problem, a hierarchical structure and pairwise comparisons are needed to describe the problem and establish relationships within the structure, respectively. The elements of the hierarchy can relate to any aspect of the decision problem once the hierarchy is built. Decision makers compare the various components placed in a column of the hierarchical matrix to the components falling in a row of the matrix and vice versa at the same time. These evaluations are then converted into numerical values that can be processed and compared over the entire range of the problem (Saaty, 1990). In this study, time-consuming nature, costliness, requirement for a specialized workforce, compliance with regulations (rules), and compatibility with the cultural environment are the five criteria should be considered in prioritizing drug shortage management strategies.

After pair-wise comparison of these criteria, a numerical weight was derived for each criterion, then these criteria were applied in second part of prioritization as the alternatives’ attributes.. Table 2 shows the results of this step.

**Table 2:** weights of criteria from AHP method

Criteria	Weights
costliness	1.064331
time-consuming nature	0.945472
requirement for a specialized workforce	1.352247
compliance with regulations	0.997908
compatibility with the cultural environment	1.012875

For the second part of prioritization, 37 alternative strategies were rated on the basis of the five criteria using the technique for order of preference by similarity to ideal solution (TOPSIS).

Hwang and colleagues developed “TOPSIS” as a solution to MCDM problems in 1981. A MCDM may be viewed as a geometric system. This means, the “m” alternatives that are evaluated by “n” attributes are similar to “m” points in an n-dimensional space. The most preferred alternative should be a point in space that has the shortest Euclidean distance from the positive ideal solution (PIS) and the longest Euclidean distance from the negative ideal solution (NIS) [47].

The procedures of TOPSIS can be defined as follows:

Drawing the decision matrix D, which consists of alternatives and criteria, described by following items, is the first step. Where A_1_,A_2_,…A_m_ are alternatives, and C_1_,C_2_,…C_n_ are criteria,x_ij_ shows the rating of the alternative A_i_ according to criteria C_j_. The weight vector W = (w_1_,w_2_,…,w_n_) is composed of the individual weights for each criterion.

$$ D={\displaystyle \begin{array}{c}{A}_1\\ {}\cdots \\ {}{A}_m\end{array}}\ \left(\begin{array}{c}{C}_1\\ {}{x}_{11}\\ {}\vdots \\ {}{x}_{m1}\end{array}\ \begin{array}{c}\cdots \\ {}\cdots \\ {}\ddots \\ {}\cdots \end{array}\ \begin{array}{c}{C}_n\\ {}{x}_{1n}\\ {}\vdots \\ {}{x}_{mn}\end{array}\right) $$

The next step is to normalize ratings by formula given bellow. By multiplying the weights of each criterion in the corresponding preferred ratings, we can obtain the weighted normalized ratings.

$$ {\displaystyle \begin{array}{l}{r}_{ij}=\frac{x_{ij}}{\sqrt{\sum \limits_{i=1}^m{x}_{ij}^2}},\mathrm{with}\ i=1,\dots, m;j=1,\dots n\\ {}{r}_{ij}=\frac{x_{ij}}{x_{i\max }},\mathrm{with}\ i=1,\dots, m;j=1,\dots n\end{array}} $$

Then, Identifying the positive ideal solutions (PIS) A^+^ and negative ideal solutions (NIS) A^−^ are derived through fallowing formulas.

$$ {\displaystyle \begin{array}{c}{A}^{+}=\left({p}_1^{+},{p}_2^{+},\dots, {p}_m^{+}\right)\\ {}{A}^{-}=\left({p}_1^{-},{p}_2^{-},\dots {p}_m^{-}\right)\\ {}{p}_j^{+}=\left(\underset{i}{\max }{p}_{ij},j\in {J}_1;\underset{i}{\min }{p}_{ij,}j\in {J}_2\right)\\ {}{p}_j^{-}=\left(\underset{i}{\min }{p}_{ij},j\in {J}_1;\underset{i}{\max }{p}_{ij},j\in {J}_2\right)\end{array}} $$

The next step is to calculate the Euclidean distances from the positive ideal solution A^+^ and the negative ideal solution A^−^ of each alternative A_i_, respectively as follows:

$$ {d}_i^{+}=\sqrt{\sum \limits_{j=1}^n{\left({d}_{ij}^{+}\right)}^2} $$

$$ {d}_i^{-}=\sqrt{\sum \limits_{j=1}^n{\left({d}_{ij}^{-}\right)}^2} $$

Then, Calculating the relative closeness ζ_i_ (Ci) for each alternative A_i_ with respect to positive ideal solution as given by:

$$ {\xi}_i=\frac{d_i^{-}}{d_i^{+}+{d}_i^{-}} $$

Finally, Ranking the alternatives according to the relative closeness. The best alternatives are those that have higher value Ci and therefore should be chosen because they are closer to the positive ideal solution.

### Sampling and data collection

The main sampling targets were decision makers and experts who have comprehensive knowledge about drug shortage management. Participants were chosen among managers or well-known members of various organizations related to the medicine’s regulation, supply and distribution, including the country Food and Drug Administration, Red Crescent logistics, Syndicate for the production of drugs and raw materials, Syndicate of drug importers, Medical Council, major health insurance organizations, Ministry of Welfare, as well as some persons expert in the field of Pharmacoeconomics and Pharmaceutical Administration. All Participants had at least 3 years of work experience in the field of pharmaceuticals.

Data were collected by using three special questionnaires. In the first phase, the questionnaire were distributed to 40 experts, out of whom 31 returned completed instruments, corresponding to a response rate of 77.5%. In the second phase, the AHP and TOPSIS questionnaires were distributed to 30 experts, out of whom 25 returned completed questionnaires, which is equivalent to a response rate of 83%.

### Questionnaire verification

For a more rigorous examination, in each phase, the initial questionnaire was scrutinized by 10 academics and policy makers who are experts or have been involved in previous drug shortage initiatives. The validity of the questionnaire, including its clarity, comprehensiveness, and relevance, was then examined in sessions held with the 10-member expert group. Last, a questionnaire that consists of five dimensions and 45 questions was finalized.

## Results

Prioritizing of Strategies for managing drug shortages have been analyzed through MCDM algorithm. Based on TOPSIS steps which described in detail in method section, after normalizing process and calculating the PIS and NIS, the distances of alternatives from PIS and NIS values were calculated. The results can be seen in Table 3.

**Table 3:** The distances of alternative, PIS and NIS values

Alternative	Di+	Di-	Ci
A 1	1.194364	1.13577	0.487427
A 2	1.14465	1.244159	0.520828
A 3	0.420742	1.999292	0.826142
A 4	1.147524	1.095706	0.48845
A 5	1.043674	1.154522	0.525213
A 6	1.182142	1.102006	0.482458
A 7	1.679556	0.721978	0.300632
A 8	0.427908	1.884946	0.814987
A 9	0.917314	1.259652	0.578627
A 10	0.243167	2.271929	0.903317
A 11	1.190888	0.972996	0.449653
A 12	1.115362	1.131639	0.503622
A 13	1.309726	0.853346	0.394507
A 14	0.737148	1.290555	0.636461
A 15	2.014484	0.518635	0.204742
A 16	1.350266	0.963075	0.416313
A 17	1.219959	0.819113	0.401709
A 18	0.272783	2.173314	0.888482
A 19	0.431618	2.108371	0.830071
A 20	0.194746	2.505842	0.927888
A 21	0.781169	1.688111	0.683645
A 22	0.71647	1.775948	0.71254
A 23	0.821415	1.223283	0.598271
A 24	0.166692	2.559009	0.938844
A 25	0.196549	2.537498	0.928111
A 26	1.427451	0.808848	0.36169
A 27	0.860576	1.45817	0.628861
A 28	0.60708	1.796926	0.747471
A 29	0.293555	2.448433	0.892941
A 30	0.707045	1.439853	0.670667
A 31	0.631996	1.615658	0.71882
A 32	1.361619	0.783839	0.365348
A 33	0.787727	1.420091	0.64321
A 34	0.270885	2.225069	0.89147
A 35	1.38661	1.21279	0.466565
A 36	0.603773	1.699446	0.737857
A 37	0.236084	2.511644	0.91408

Then, Ci of each alternative strategy was calculated. Tables 4 present alternatives’ Ci in each dimension. In addition, strategies are ordered from largest to smallest Ci in Table 5.

**Table 4:** The alternatives’ Ci in each dimension

Dimensions	Related alternatives	Ci (rank of TOPSIS)
**Regulatory**	Alternative 1	0.487427
	Alternative 2	0.520828
	Alternative 3	0.826142
	Alternative 4	0.48845
	Alternative 5	0.525213
	Alternative 6	0.482458
	Alternative 7	0.300632
	Alternative 8	0.814987
	Alternative 9	0.578627
	Alternative 10	0.903317
**Financial**	Alternative 11	0.449653
	Alternative 12	0.503622
	Alternative 13	0.394507
**Supply Chain (SC)**	Alternative 14	0.636461
	Alternative 15	0.204742
	Alternative 16	0.416313
	Alternative 17	0.401709
	Alternative 18	0.888482
	Alternative 19	0.830071
	Alternative 20	0.927888
	Alternative 21	0.683645
	Alternative 22	0.71254
	Alternative 23	0.598271
**IS**	Alternative 24	0.938844
	Alternative 25	0.928111
**Policy-making**	Alternative 26	0.36169
	Alternative 27	0.628861
	Alternative 28	0.747471
	Alternative 29	0.892941
	Alternative 30	0.670667
	Alternative 31	0.71882
	Alternative 32	0.365348
	Alternative 33	0.64321
	Alternative 34	0.89147
	Alternative 35	0.466565

**Table 5:** TOPSIS rank of total strategies

#	Priorities	Dimension	sorted Ci	#	Priorities	Dimension	sorted Ci
1	A 24	IS	0.933745	20	A 27	Policy	0.598001
2	A 20	SC	0.933213	21	A 23	SC	0.582914
3	A 25	IS	0.919914	22	A 9	Regulatory	0.568572
4	A 37	Policy	0.918045	23	A 5	Regulatory	0.498484
5	A 18	SC	0.893702	24	A 6	Regulatory	0.48755
6	A 29	Policy	0.89023	25	A 12	Financial	0.469364
7	A 34	Policy	0.889904	26	A 2	Regulatory	0.462392
8	A 10	Regulatory	0.888378	27	A 4	Regulatory	0.456985
9	A 19	SC	0.844232	28	A 11	Financial	0.43357
10	A 8	Regulatory	0.811773	29	A 1	Regulatory	0.431961
11	A 3	Regulatory	0.81176	30	A 13	Financial	0.423617
12	A 28	Policy	0.73262	31	A 16	SC	0.41103
13	A 36	Policy	0.721464	32	A 17	SC	0.403283
14	A 22	SC	0.7037	33	A 35	Policy	0.399494
15	A 31	Policy	0.698801	34	A 26	Policy	0.380528
16	A 21	SC	0.661945	35	A 32	Policy	0.368989
17	A 30	Policy	0.639332	36	A 7	Regulatory	0.321964
18	A 33	Policy	0.620863	37	A15	SC	0.219713
19	A 14	SC	0.610579				

Table 5 shows that strategies 24 and 25, which are related to the information system (IS) dimension, and strategy 20, which belongs to the supply chain dimension, were accorded high priority by the experts. The creation of IS for visibility or information sharing among supply chain entities is one of the critical elements of an effective supply chain [48], and can aid appropriate predictions of shortages. In the pharmaceutical supply chain, there is a growing need for the pharmaceutical industry to secure their distribution channels against the proliferation of counterfeit drugs, and the healthcare system is currently looking for improved methods of monitoring their pharmaceuticals not only along the supply chain but also once prescriptions have been filled. This is where IS comes in. The aforementioned requirements are currently being satisfied with the help of radio-frequency identification (RFID) systems. RFID solutions are ideal identification methods by which the pharmaceutical industry can combat issues associated with fake drugs and improve quality, reduce costs, and most importantly, improve patient safety issues given the capability of RFID technology to capture and transmit data [49].

To define prominent dimensions, the ratio of number of strategies with Ci greater than 0.5 to the all number of strategies in each dimension were calculated.

The results are presented in Table 6, which shows that IS was accorded 100% priority. Following IS in order of importance are the policy and supply chain dimensions. The dimensions of low importance from the perspectives of the experts are regulatory and financial issues.

**Table 6:** Priority of dimensions

Dimension	Total strategies	strategies Ci > 0.5	%
SC	10	7	70%
Policy	12	9	75%
Financial	3	0	0.0%
Regulatory	10	4	40%
IS	2	2	100%

## Discussion

Healthcare systems rely on a consistent supply of pharmaceuticals to support patient care. The purposes of this study were to identify (first phase) and prioritize (second phase) the strategies that healthcare policymakers use to effectively address and manage supply shortages. Thirty-seven strategies were identified under regulatory, financial, supply chain, information system, and policymaking dimensions as the most important approaches to drug shortage management. The prioritization of these strategies was determined on the basis of five main indicators, namely, time-consuming nature, costliness, the requirement for a specialized workforce, compliance with rules, and compatibility with the cultural environment. The results showed that the IS, policymaking, and supply chain dimensions are top priority issues.

The IFDA’s definition of shortage is similar to that of the FDA. However, shortage data collected in Iran through the IFDA’s Information Center of Medicine showed that the definitions are not accepted by Iranian physicians and patients; they refuse to implement drug substitutions and most of the time seek original branded medicines [3]. Certain suppliers also violate IFDA regulations, and the intensive inventory control meetings are sometimes inadequate measures for addressing scarcity. That is, suppliers do not conform to predetermined supply programs, thereby causing drug shortages. A number of other problems plague the Iranian healthcare system. Despite data collection through routine market monitoring in the country, the warning system of the IFDA’s Information Center of Medicine does not suffice as a comprehensive system [45]. Based on implicit findings derived from Cohen et al. (2013) [6], Ventola et al. (2011) [12], and Fox (2014) [23] studiesthe warning system of Iran should be upgraded to an advanced structure through enhanced collaboration among stakeholders, the publication and enforcement of clear national guidelines for drug shortage management, and the improvement of the IFDA’s capability to exercise control over the pharmaceutical market. These recommendations are in line with the current research’s findings and confirm that an enhanced IS and policy infrastructure can help the IFDA more effectively prevent and manage drug shortages. Finally, American Society of Health-System Pharmacists (ASHP) [19] and the FDA [3] asserted that the lack of IT-based threat analysis, risk assessment, and documentation in Iran weakens the IFDA’s capabilities to determine the potential consequences of a shortage and effectively set policies. To prevent this problem, we proposed creating different databases at different levels and using them to prevent, response, and recovery phases of drug shortage in order to have appropriate communication, and accurate and correct flow of information between related parties, within the IFDA departments, and from the IFDA to the population. So, it is important as a measure to increase the interest of stakeholders (especially patients) in drug shortages issue through the social media. In this regard, unnecessary anxiety, such as when people are exposed to excessive emphasis or incorrect information must be considered.

As previously stated, the IFDA implements a distribution policy during shortage events, and evidence has shown that patients are prioritized when drug shortages occur in the country [42]. According to Tyler (2002), ASHP (2009), and Ventola et al. (2011) [12, 19, 39], this procedure can help eligible patients adhere to their treatments, and less eligible patients avail of substitutions. In this regard, clear policies can help a healthcare system manage drug shortages successfully. Patient prioritization requires the collection of patient information in advance, which in turn, necessitates the construction of an IS infrastructure that functions all over a country and the formulation of excellent enforcement policies.

The findings of the current research support those obtained in some previous studies. As Steinbrook et al. (2009) and Fox (2014) [16, 23] demonstrated, effective SCM approaches can minimize the possibility of drug shortage occurrence. At the IFDA level, considering strategic stocking and multi-supply sources, especially with respect to imported medicines, can be regarded as policy and SCM issues. Tyler and Pharm (2002), Johnson et al. (2011) [1], and Ventola et al. (2011) [12] stated that analyzing prescription trends and behaviors, applying alternative therapies, and increasing awareness about shortages can help the IFDA manage these situations more effectively. The Rational Use of Medicine (RUD) and Drug and Poisons Information Center (DPIC) constitute an acceptable IFDA infrastructure for dealing with such issues, but these organizations require efficient policies and IT facilities.

Most interventions that can reduce the incidence of drug shortages can be established on the basis of regulations. Although the results of this research showed that regulatory issues are of fourth-level priority in shortage management, drug supply shortages occur for various reasons and backgrounds, and the priority of avoidance and response measures is expected to differ depending on the reasons and backgrounds. For example, if the drug was recalled and withdrawn from the market due to a defect in the manufacturing process, that regulation (investigation of the cause and countermeasures) would be given higher priority.

Considering the role of policies as upstream components from which to draft related regulations, the existence of clearly stated and comprehensive policies can automatically engender good regulations. Financial issues were accorded the least priority in this research—a finding that may be attributed to the overall financial situation of Iran. This prioritization can, nevertheless, rapidly change when financial components are altered.

Guimaraes et al. (2013) revealed that particular partnerships among parties within a supply chain may foster information sharing, which can decrease information gaps and data fragmentation within management reporting applications in a distribution channel [50]. From the perspective of information gathering, drug shortage management may drive diligent efforts to maintain information integration [51].

The importance of adopting appropriate and comprehensive policies is undeniable. In this regard, Sharma et al. (2013) explained that the globalization of modern treatment protocols places an additional burden on supply chains and distribution channels in their efforts to maintain an adequate supply of critical medications [52]. Heydari et al. (2015) concluded that intensive care nurses should receive specialized training to curtail supply chain disruptions and other training programs on the practical management of a hospital’s scarce resources. In this respect, a general problem is that some pharmaceutical policymakers address operational challenges resulting from suboptimal pharmaceutical supply chain disruptions [53]. A specific problem is that some pharmaceutical procurement leaders have limited strategies for addressing disruptions to drug supply chains and distribution channels.

1. Jahanbakhsh et al. (2013) [54] and Mehralian et al. (2015) [55] showed that transitioning from a traditional push system supply chain to a more efficient pull system enables better workforce utilization and cost control than do traditional methods; with the changes stimulated by the development of organizational agility or process fitness, drug shortages and the uncertainty and unpredictability that arise from its nature can be managed more efficiently.

## Conclusion

Base on the results, strategies related to IS, policy-making, and SC are the most important in sustainable drug shortage management. As health systems depend on sustained pharmaceuticals supplies to manage patients, the policymakers should try to share information and provide conditions for transparent access to information for all healthcare stakeholders. Therefore, creation of IS to make visibility or information sharing among supply chain entities is a critical element for having an effective SC and can help to predict and manage the shortage properly.

Finally, by improving data gathering method in Iran through better collaboration with stakeholders, publishing the national guidelines for sustainable drug shortage management, and enforcing the guidelines can help the IFDA to improve own shortages management capability and overcome to the shortage crisis.

## Managerial implications, limitations, and recommendations for future research

This paper identified and prioritized five dimensions related to strategies that may effectively advance drug shortage management in Iran. On the basis of the results, the following practical recommendations were formulated:

- The definition of drug shortage and the policies that underlie its management should be reviewed on the basis of community expectations.
- Community awareness and health literacy on scientific substitutions during shortages should be increased and promoted, and guidelines in this regard should be enforced.
- Effective punishments for the failure of stakeholders to commit to a supply program should be imposed.
- Experiences with drug shortages should be documented.
- An advance warning system should be developed using IT-based mandatory updated rolling forecast.
- Strategic stocking should be applied.
- Intensive inter-organizational collaboration (central bank, customs, national pricing committee, insurance organisations, etc.) should be exercised in addressing drug shortages in accordance with priorities.
- A track-and-trace system for monitoring the supply chain, prioritizing patients, and implementing effective distribution based on historical distribution patterns during shortages should be created.
- RUD guidelines for physicians should be enforced.
- A watchdog system should be designed to monitor pharmaceutical markets around the country continuously.

These important suggestions may influence business practices for strategic planning in pharmaceutical companies; healthcare procurement leaders can also contribute their new knowledge in the development of strategies for addressing drug shortages. Policymakers may use the findings as reference for assessing key strategies for delivering pharmaceutical products from manufacturers to end-users.

Similar to any other study, the current work also has certain limitations, primary among which revolves around the sample. The study included decision makers and experts as internal stakeholders in the healthcare system of Iran. Although external stakeholders such as the customs bureau are also affected by shortages, the research did not explore the involvement of these parties. In the future study, the impact of external stakeholders can be examined on incidence of drug shortages and their management.

Scholars, legislators, decision makers, and industry leaders have been unable to provide comprehensive solutions to the issue of drug shortage. Much of the focus of explorations into this problem concentrated on ex post facto strategies that are implemented to help healthcare providers mitigate the effects of drug supply disruptions. The adaptive nature of the complex and chaotic system known as pharmaceutical supply and distribution requires systematic approaches to understanding the dynamics that govern drug shortages.

## Data Availability

The datasets used and/or analyzed during the current study available from the corresponding author on reasonable request.

## Abbreviations

AHP: Analytic Hierarchy Process
API: Active Pharmaceutical Ingredient
ASHP: American Society of Hospital Pharmacies
Ci: Relative Closeness Index
DPIC: Drug and Poisons Information Center
FDA: Food and Drug Administration
IFDA: Iran Food and Drug Administration
IS: Information System
MCDM: Multiple Criteria Decision Making
NDPIC: National Drug and Poisons Information Center
PIS: Positive Ideal Solution
NIS: Negative Ideal Solution
NRUD: National Rational Use of Medicine
RFID: Radio-frequency Identification
RUD: Rational Use of Medicine
SC: Supply Chain
SCM: Supply Chain Management
TOPSIS: Technique for Order of Preference by Similarity to Ideal Solution
